# Acute kidney injury applying pRifle scale in Children of Hospital Universitario del Valle in Cali, Colombia: clinical features, management and evolution

**Published:** 2012-09-25

**Authors:** Consuelo Restrepo de Rovetto, Julián A Mora, Sergio Alexandre Cardona, Andrés F Marmolejo, Juan F Paz, Iris de Castaño

**Affiliations:** a Departamento de Pediatría, Escuela de Medicina, Facultad de Salud, Universidad del Valle. Cali, Valle del Cauca, Colombia. E-mail: rovettos@gmail.com, ircastan@yahoo.com; b Escuela de Medicina, Facultad de Salud, Universidad del Valle, Cali, Valle del Cauca, Colombia. E-mail: mora.uv@gmail.com

**Keywords:** Acute Kidney Injury, classification, pRIFLE, Etiology, sepsis, children

## Abstract

**Objective:**

To know the epidemiology of Acute Kidney Injury (AKI) in the pediatric population at Hospital Universitario del Valle (HUV), a tertiary University Hospital in Cali, Colombia.

**Methods:**

We obtained a series of cases through daily surveillance for a seven-month period (June 1 to December 31, 2009) in patients older than 30 days and under 18 years at HUV. We excluded patients with previous diagnosis of chronic renal failure. The new pRIFLE scale was used to define AKI.

**Results:**

27 patients were detected, with mean age of 36 months. Incidence of AKI was 0.38% from pediatric admissions and 6.2% from the pediatric intensive care unit (pICU) admissions. The pRIFLE scale at study entrance was: Risk: 2 patients, Injury: 8, Failure: 17. Etiology of AKI was: pre-renal in 89%, primary renal disease in 3.7%, and post-renal in 7.4%. There was an association of AKI with sepsis in 66.7% and 48.2% progressed to septic shock. Six patients required renal replacement therapy, all required peritoneal dialysis. The AKI was multi-factorial in 59.3% and associated with systemic multi-organ failure in 59.3%. At study entry, 63% patients were in pICU. The average hospital stay was 21.3 ± 9.2 days. Six children died, 16 resolved AKI, and nine were left with renal sequelae.

**Conclusions:**

We recommended pRIFLE scale for early diagnosis of AKI in all pediatric services. Education in pRIFLE scale, prevention of AKI, and early management of sepsis and hypovolemia is recommended.

## Abstract (es)

**Objetivo:**

Conocer la epidemiología de la injuria renal aguda en la población pediátrica en el Hospital Universitario del Valle (HUV), un hospital de referencia de III nivel en Cali, Colombia.

**Metodos:**

Se obtuvo una serie de casos por medio de vigilancia diaria por un periodo de 7 meses (1 de Junio a 31 de Diciembre del 2009), en pacientes mayores de 30 días y menores de 18 años del HUV. Se excluyeron pacientes con diagnóstico previo de insuficiencia renal crónica. Para definirla IRA se utilizó la escala pRIFLE.

**Resultados:**

Se detectaron 27 pacientes, con una mediana de edad de 36 meses. La incidencia de IRA fue 0.38% de las admisiones de pediatría y 6,2% en la unidad de cuidado intensivo pediátrico (UCIp). El pRIFLE al ingreso del estudio fue: Riesgo 2 pacientes, Injuria 8, Falla 17. La etiología de la IRA fue pre renal en el 89%, enfermedad renal primaria 3.7% y 7.4% post renales. La IRA se asoció a sepsis en 66.7% y evolucionaron a shock séptico 48,2%. Seis pacientes requirieron terapia de remplazo con diálisis peritoneal. La IRA fue multifactorial en el 59.3% y estuvo asociada a falla orgánica multisistémica en 59.3%. Al ingreso del estudio 63% se encontraban en UCIp. Fallecieron 6 niños, resolvieron la IRA 16, quedando 9 con secuelas renales.

**Conclusiones:**

Se recomienda aplicar la escala pRIFLE para el diagnóstico temprano en todos los servicios pediátricos. Se deben implementar educación en la escala de pRIFLE, prevención y manejo temprano de sepsis e hipovolemia.

## Introduction

Acute kidney injury (AKI) is a pathology defined as a sudden loss of renal function that can be reversible. Previously, no standard definition of AKI existed, which hindered comparison among studies^1^. The RIFLE scale, proposed in 2004 by Bellomo^2^for early detection and classification of the AKI severity is currently the most widely accepted; RIFLE is an acronym where each letter means a level of severity of AKI (R = risk, I = injury, F= failure, L= loss E= end-stage kidney disease). The definition is based on two criteria: decreased rate of glomerular filtration estimated based on serum creatinine and/or the duration of oliguria or anuria. The scale also permits establishing AKI severity. After applying the scale on adults, its validation was conducted on children and modified as the pediatric scale or pRIFLE^3^ (Table 1) this scale was used to define AKI in this study.

**Table 1:**
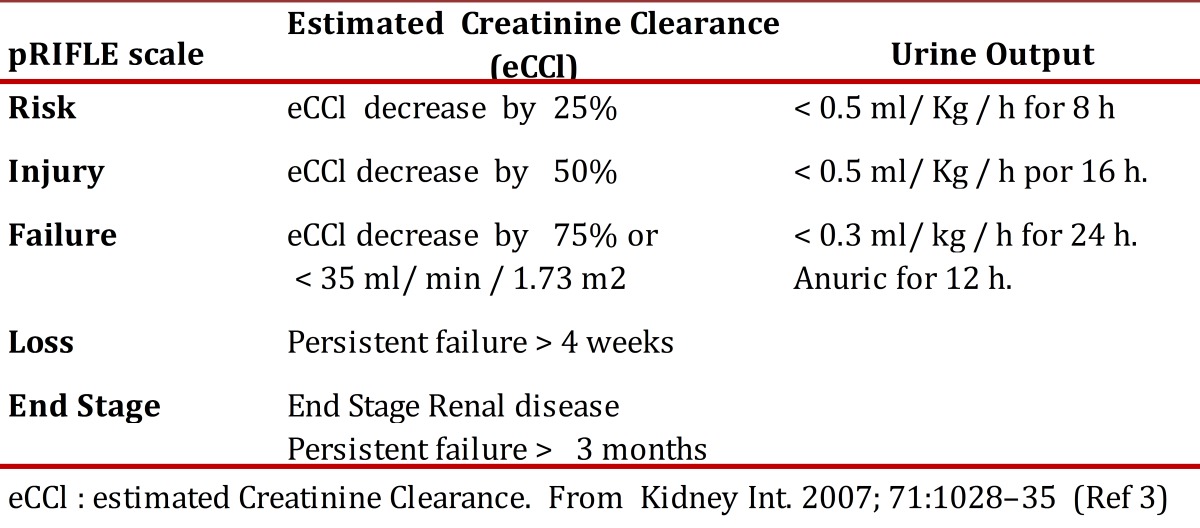
pRIFLE Criteria for Clasification of Acute kidney Injury in children. eCCl : estimated Creatinine Clearance. From Kidney Int. 2007; 71:1028-35 (Ref 3)

Studies of AKI in pediatric patients show that the causes and incidence of AKI depend on the country's level of development, the hospital's level of complexity, and on the definitions used. The incidence is not clear due to the multiple definitions used in the different studies^1^. Most of the studies published on AKI were conducted on patients hospitalized in pediatric intensive care units (pICU).

Application of the pRIFLE Scale on patients in the pICU permits detecting earlier forms of AKI, evaluating its severity, and predicting the hospital stay, mortality, and need for renal replacement therapy in pediatric patients^3^ It is considered that the pRIFLE scale started a new era in the study of AKI^4^ in children, given that it permits a standard definition with which multi-centric studies may be carried out, associated risk factors may be evaluated, along with the need for renal replacement therapies, and evolution of patients according to AKI severity.

With the new pRIFLE classification, it has been detected that AKI incidence is higher than previously described^5^ When reviewing AKI epidemiology in children, during recent years, by applying the pRIFLE Scale in patients in the pICU, it ranges from 35.9to 85%, if early stages of AKI are included^5^

Hospital Universitario del Valle has a total of 576 beds, of which 83 are for pediatric hospitalization, 11 for pediatric intensive care, 27 for intermediate neonatal care, and 12 for neonatal intensive care, which is why it is vitally important to have data pertaining the epidemiology of this pathology in this hospital center.

The aim of this study was to describe the epidemiological profile of acute kidney injury (AKI) in the pediatric population at Hospital Universitario del Valle (HUV).

## Materials and Methods

### Design:

A series of cases were obtained of patients with AKI via daily surveillance between 01 June and 31 December 2009.

### Population:

The investigation included patients older than 30 days and younger than 18 years of age, with diagnosis of AKI, located in pediatric wards at HUV, including the pediatric emergency service. Those with prior diagnosis of chronic kidney insufficiency, as well as those who refused informed consent were excluded.

### Procedure:

Before starting the study, the protocol was transmitted throughout the different hospitalization services. Daily surveillance took place at the pediatric wards in HUV, during a seven-month period, searching for patients with AKI according to the criteria of the pRIFLE Scale^3^. Informed consent was requested from the child's parents or legal guardian.

From the clinical history during hospitalization and in the format designed for the study, socio-demographic variables were collected, along with the pRIFLE Scale on admissions, AKI clinical data, treatment received, and patient evolution. Height was obtained from the clinical history, or when necessary, patients were measured to calculate the estimated rate of glomerular filtration. Each patient was monitored from the moment of entry in the study until the resolution of the AKI or for a maximum 30-day period. Information from the forms was recorded on the Epi-Info program version 3.5.1. Univariate analysis was performed to characterize the study population, describing the categorical variables via central tendency and dispersion measurements.

The study was approved by Human Ethics Committees in the Faculty of Health at Universidad del Valle and HUV.

## Results

During the seven-month surveillance, 28 patients were detected with AKI; informed consent was refused in one of them, leaving 27 for analysis.

Data from the patients detected is summarized in Table 2, in the order in which they entered the study. A total of 52% were females, mean age was 54.22 months. The diagnoses were taken as they were reported in the clinical history.Some 55.6% presented AKI during hospitalization. Upon diagnosis, 63% of the patients were in the pICU, 18.5% in the emergency service, and 18.5% in hospitalization wards.

**Tabla 2:**
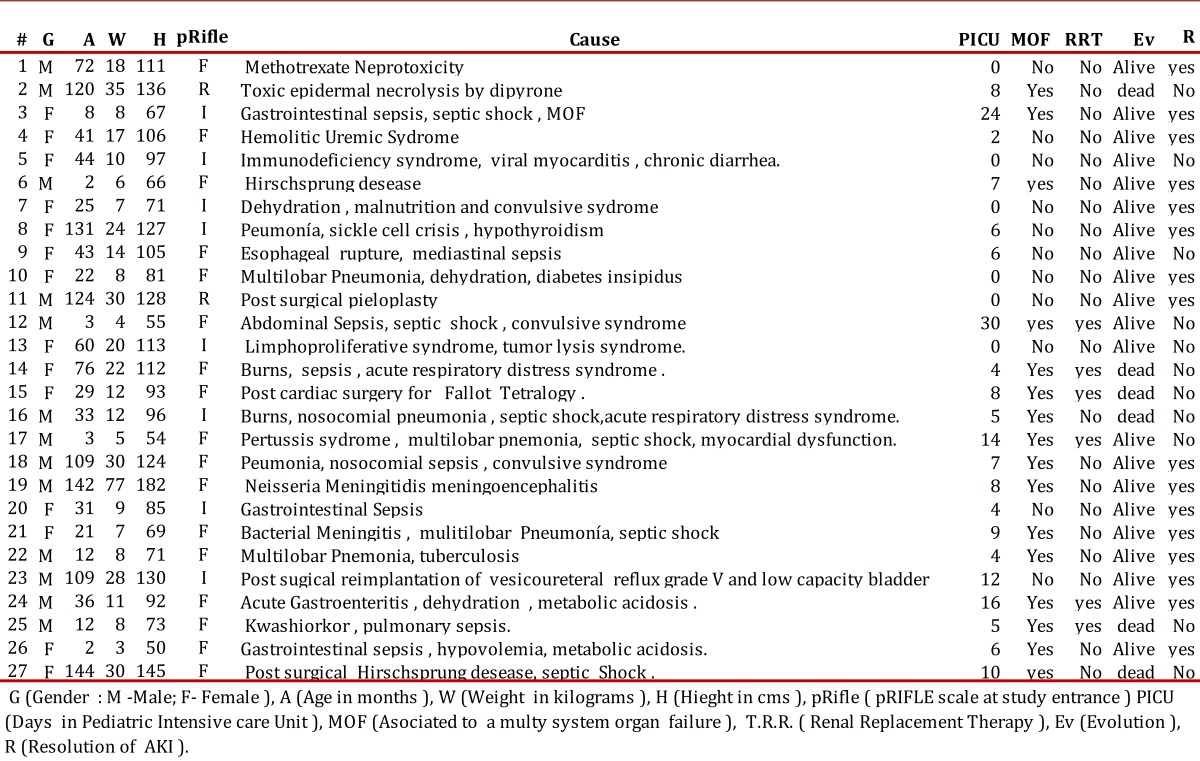
Summary of pediatric patients with Acute Kidney injury al HUV, 2009. G (Gender : M -Male; F- Female ), A (Age in months ), W (Weight in kilograms ), H (Hieght in cms ), pRifle ( pRIFLE scale at study entrance ) PICU ( Days in Pediatric Intensive care Unit ), MOF (Asociated to a multy system organ failure ), T.R.R. ( Renal Replacement Therapy ), Ev (Evolution ), R (Resolution of AKI ).

The incidence, stage of pRIFLE upon entering the study, the clinical classification, causality, and evolution of the AKI are summarized in Table 3.

**Table 3:**
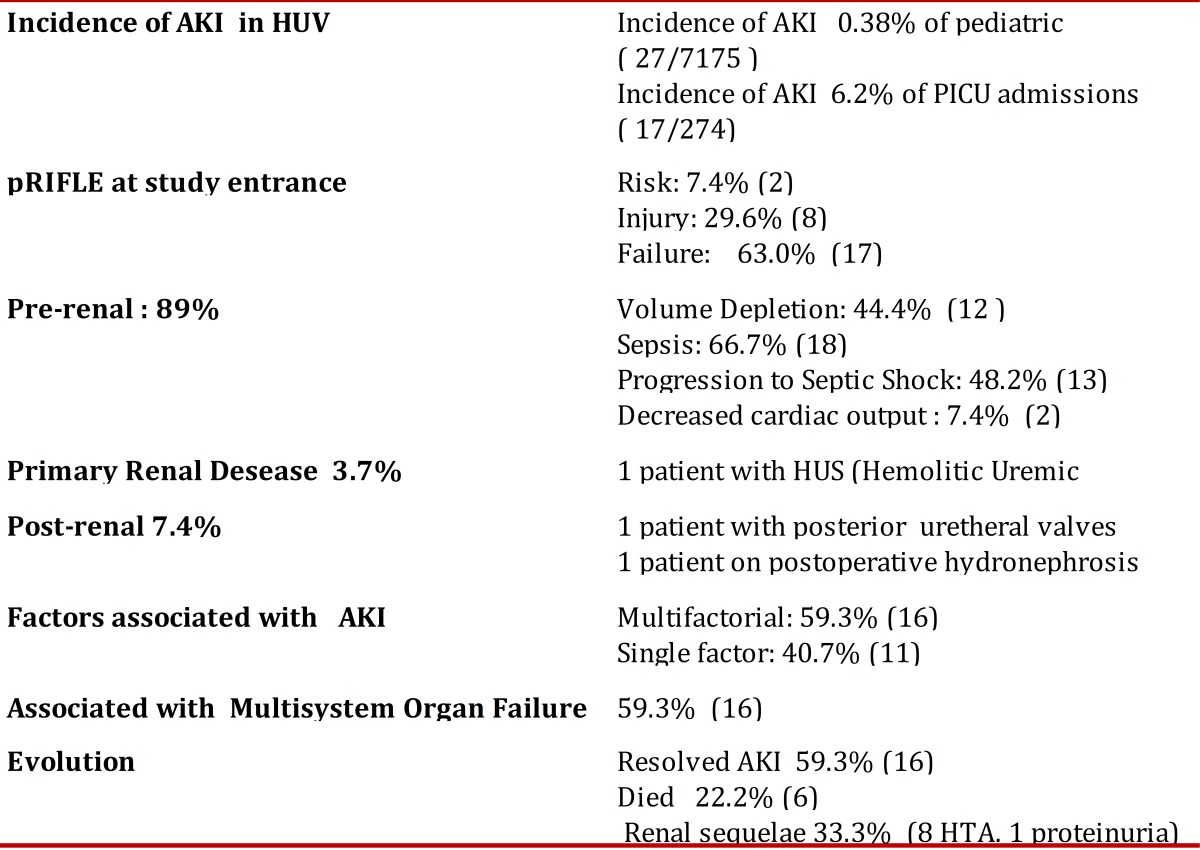
Incidence, classification and evolution of pediatric patients with AKI, HUV 2009, N= 27

The AKI incidence of all the hospitalizations was at 0.38% and of the patients in pICU it was at 6.2%. The pRIFLE upon entering the study was of Failure in 63% of the cases. 

The etiology of AKI revealed predominance of pre-renal causes (89%) with prevalence of sepsis and volume depletion. The AKI was multi-factorial in 59% of the cases.A total of six patients died (22.2%); all with sepsis, multi-factorial renal failure, and multi-systemic organic failure.

The clinical presentation of AKI was nonoliguric in 48.2%, oliguric in 44.4%, and anuric in 7.4%. Additional symptoms of AKI were: edema in 22.2%, metabolic acidosis 22.2%, hypotension on admission 18.5%, encephalopathy 14.8%, hypertension 44.4%, and hypovolemia 40.7%.

Table 4 summarizes the medical treatment offered to the patients and the hospital stay. Six children received renal replacement therapy with peritoneal dialysis; of these, three died. No complications associated to peritoneal dialysis became evident.

**Table 4:**
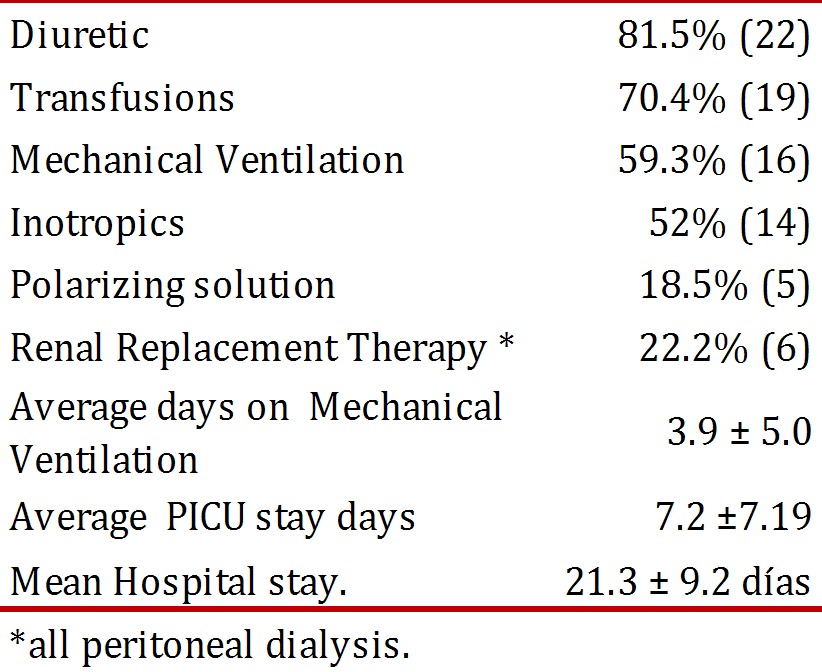
Medical therapy offered to pediatric patients with AKI and hospital stay, HUV 2009, N= 27

Some 52% of the patients were from Cali and Valle del Cauca (<100 km from the hospital); 37% from Cauca (>100 km), and 11% from other departments (>200 km).

It was reported that 74% of the patients had subsidized social security scheme, 22.2% without insurance, and 3.7% had contributive healthcare. Nearly 92.6% belonged to socio-economic level 1 and 7.4% to level 2. Regarding ethnicity, 40.7% were Mestizo, 40.7% Afro-descendants, 14.8% Indigenous, and 3.7% Caucasian.

## Discussion

The incidence of AKI varies according to the population studied, the level of attention of the hospital center, and the country's level of development^1^. Today, the tendency is to perceive AKI as an evolutionary spectrum and classify it with scales of severity or stages of AKI like the pRIFLE Scale, which was validated in 2007^3^. It has been shown that the incidence of AKI increases when applying the pRIFLE Scale^3^ ^5^.

In this study, epidemiological surveillance of AKI was conducted in the Pediatric services at HUV applying the pRIFLE Scale. The strength of this study lies in that the epidemiological surveillance was not limited to the unit of pediatric care, like most studies, but that it took place in all pediatric services, permitting us to know the magnitude of this pathology in this reference hospital center (Table 3).

The incidence of AKI in the pICU was 16 times higher than in wards, which shows that the risk of AKI increases as the patient is most critical; however, the active search or surveillance of this pathology in emergency services and wards is important.

By applying the pRIFLE Scale, prospective studies have been conducted; the greatest number of cases was a multi-centric study in Turkey^6^ where 472 children were evaluated: 154 neonates and 318 children older than one month, in 17 nephrology centers, for a one-year period. Comparing the pRIFLE stages at the moment of diagnosis between the study in Turkey^6^ and that at HUV, we have that 30.8% vs. 7.4% were detected at Risk; 25% vs, 29% in Injury; and 43% vs. 63% in Failure. This reflects that diagnosis during the early stages of AKI, where therapeutic interventions can be offered to stop AKI progress, is low in our center. At HUV, 44.4% of the cases presented AKI upon admission to the hospital; this could explain the low detection of AKI in the Risk stage and the complexity of patients referred.

In Colombia, few studies have been conducted on the etiology of AKI in children. In a 10-year retrospective study (1996-2006), carried out at Fundación Valle del Lili (FVL) in Cali^7^, 2.1% prevalence of AKI in the pICU was found, using serum creatinine above 1.5 mg/dL as definition of AKI. A second prospective study (2009-2010) in this pICU, applying the pRIFLE Scale^8^ found increased incidence at 4.9%. In our current study, the incidence of AKI in pICU was 6.2%, somewhat higher, which could be due to the number of critical patients referred to the HUV.

Upon analyzing the AKI etiology, pre-renal causes predominated, as described in other studies^9^ Sepsis and volume depletion were the main causes of pre-renal failure. The percentage of patients with sepsis at HUV is at 66.7%, above that in other studies: 28.7% in the study conducted in Nigeria^9^ 27% from the study at FVL^8^, and 15.5% in the multi-centric study in Turkey^6^ This could be explained by a number of factors: the low socio-economic level of the patients cared for at HUV, which could have nutritional implications, the high level of referrals of patients from Valle del Cauca and other departments, and the fact that at HUV cardiac surgery or transplants are not performed. Post-surgical cardiac patients, hematology/oncology conditions, ischemia, and nephrotoxicity are main causes of AKI in other studies^6^and at FVL^8^.

This series of cases two patient with skin burns was noted, who both died with sepsis and multi-systemic organic failure. In a retrospective study to assess AKI in burnt pediatric patients^10^ using the pRIFLE Scale, AKI incidence was at 45.5%; patients with AKI had higher mortality and sepsis contributed to developing pRIFLE in Failure.

Of the 18 patients with sepsis in this case series, 13 evolved to septic shock. In adults, AKI occurs in 19% with moderate sepsis, in 23% with severe sepsis, and 51% with septic shock^11^. Mortality of patients who have AKI with sepsis is at 70%, compared to 45% in those who have only AKI^11^. The combination of sepsis and renal failure increases the risk of mortality, which has been ratified in multiple studies, both in children^6^ ^10^ and adults^11^ ^,^ ^12^.

Use of inotropic agents has been considered a prognostic factor of AKI^1^ ^,^ ^9^. In 18.5% of the cases, hypotension was noted upon entering the study and 52% of the patients received inotropic agents within the medical management, suggesting some type of shock, which increases risk of AKI^12^.

Acute kidney injury due to primary renal disease was low at 3.7% (one patient with hemolytic-uremic syndrome), possibly due to the number of cases and the time of surveillance; however, recent studies^6^ show that with the development of medicine and the support to patients in critical state, AKI increasingly occurs more because of systemic disease than because of primary renal pathologies.

Support with renal replacement therapy must be done early to improve patient survival^1^. The pRIFLE Scale helps to define criteria to start timely renal support therapy in patients with AKI^6^ ^,^ ^13^. In a prospective study^6^with 423 cases of pediatric AKI, requirements for dialysis according to the pRIFLE Scale were: 10% for Risk, 25.7% for Injury, and 48.9% for Failure. In our case series, all requiring peritoneal dialysis revealed Failure in the pRIFLE scale.

Other studies have shown that mortality of patients with AKI is higher with more severe patient pRIFLE scores^2^ ^3^ ^10^ ^12^. In the current study, patient mortality was also associated to the pRIFLE Scale, of the six patients who died four (66.7%) had pRIFLE F and (16.7%) pRIFLE I, agreeing with the literature.

Of the 27 patients participating in the study, nine (33%) developed renal sequelae; patients with AKI must be controlled in the long term and in outpatient manner, given the risk of proteinuria, hypertension, and diminished renal function.

Mean age was 36 months, showing that infants and pre-school children have an important risk of developing AKI, similar to that described in the study at FVL^8^ with 24 months and 34.8 months in the study in Turkey^6^. Other studies have reported higher mean ages (6 - 7 years) ^9^ ^,,^ ^13^; these age differences may be because these are retrospective studies reviewing the clinical histories of patients referred to pediatric nephrology centers, with different socio-demographic characteristics.

In this case series, 59.3% (16) required ventilator support, with average days on the ventilator of 3.9 ± 5.0; it has been noted that mechanical ventilation is a predictor of mortality in critical patients with AKI^6^ with RR of 8.731 with a 95% confidence interval (*p*< 0.001).

The number of patients requiring transfusions was high with 70.4% showing the critical state of these patients. The presence of edema, metabolic acidosis, and hypertension was similar to that reported in other studies^13^.

The retrospective study of clinical histories in Turkey^13^, found that AKI was multi-factorial in 31% of the cases, compared to 59.3% from the current study, which shows the complexity of the patients seen at HUV.

Comparing this study with that in Nigeria^9^, the clinical presentation of AKI was: nonoliguric in 48.2% vs. 16.3%. Use of diuretics was documented in 81.5% of the cases, which may explain the high percentage of nonoliguric renal failure.

The average hospital stay was of 21.3 ± 9.2 days and the average stay in the pICU was of 7.2 ± 7.19, which evidences the critical state and the costs implied of patients with AKI.

The socio-economic level of patients cared for at HUV is low with 92.6% from level 1; additionally, only 3.7% have a contributive social security scheme. Patients seen at HUV have scarce economic resources and lower social security coverage. The socio-economic level and availability of healthcare services contribute to the etiology and prognosis of AKI, which has been demonstrated in other studies^1^ ^,^ ^6^ ^,^ ^13^.

Regarding patient background, 48% were not from Valle del Cauca: 37% were from Cauca and 11.1% from other departments (>200 km from the hospital), which shows that the HUV is a reference center for the Colombian southwest and its regions.

In terms of ethnicity, an equal percentage was found of Mestizo and Afro-descendants, with an indigenous population at 4%, which corresponds to the departments of origin.

Among the strengths of this study, we have that it was conducted in surveillance manner, which permitted determining the incidence of AKI, was not limited to patients in the pICU, and used a new scale for its diagnosis. Information was obtained daily from the clinical history at bedside. Among the weaknesses is that it is a case series collected at a reference hospital from the Colombian southwest that takes in very critical patients with poor coverage of medical services.

This study and multiple studies^3^ ^,^ ^5^ ^,^ ^6^ ^,^ ^8^ ^,^ ^10^ in children, ratify the usefulness of the pRIFLE scale for early diagnosis, classification, and prognosis of AKI. Surveillance of this pathology must be maintained in the pICU, hospitalization, and emergency services.

The study permitted educating the healthcare personnel at HUV in this new classification. Education strategies must be implemented in the pRIFLE Scale, prevention of AKI, prevention of sepsis and hypovolemia, early management of AKI, and timely referral or inter-consultation of patients at risk of AKI. 

Future multi-centric pediatric studies of AKI, applying this new pRIFLE Scale and with standardized management of AKI will permit greater information on this pathology and will determine the risk factors in children. 

Clinical implementation of new urinary biomarkers^1^ ^,^ ^5^ for early diagnosis of AKI during the Risk stage will permit early therapeutic intervention and will improve prognosis of children and adults with AKI.
